# NiO Nano- and Microparticles Prepared by Solvothermal Method—Amazing Catalysts for CO_2_ Methanation

**DOI:** 10.3390/molecules29204838

**Published:** 2024-10-12

**Authors:** Arkadii Bikbashev, Tomáš Stryšovský, Martina Kajabová, Zuzana Kovářová, Robert Prucek, Aleš Panáček, Josef Kašlík, Tamás Fodor, Csaba Cserháti, Zoltán Erdélyi, Libor Kvítek

**Affiliations:** 1 Department of Physical Chemistry, Faculty of Science, Palacky University Olomouc, 17. listopadu 12, CZ-77146 Olomouc, Czech Republic; arkadii.bikbashev01@upol.cz (A.B.);; 2 Czech Advanced Technology & Research Institute CATRIN, Regional Centrum of Advanced Technologies & Materials, Palacký University Olomouc, Slechtitelu 27, CZ-78371 Olomouc, Czech Republic; 3 HUN-REN Institute for Nuclear Research, H-4002 Debrecen, Hungary; 4 Department of Solid-State Physics, Faculty of Sciences and Technology, University of Debrecen, H-4002 Debrecen, Hungaryzoltan.erdelyi@science.unideb.hu (Z.E.)

**Keywords:** nickel oxide, solvothermal synthesis, morphology, carbon dioxide, methanation

## Abstract

Nickel oxide (NiO) is one of the most popular hydrogenation catalysts. In heterogeneous catalysis, nickel oxide is used, for example, as a suitable methanation catalyst in the Fischer–Tropsch reaction not only for CO hydrogenation but also in the modified Fischer–Tropsch reaction with CO_2_. However, CH_4_ selectivity and CO_2_ conversion strongly depend on NiO micro- (MPs) and nanoparticles’ (NPs) shape, size, and surface area. In this study, the synthesis of NiO micro- and nanoparticles was conducted using the simple solvothermal method. Different morphologies (microspheres, sheet clusters, hexagonal microparticles, and nanodiscs) were prepared using this method with different solvents and stabilizers. The prepared catalysts were tested in the hydrogenation of CO_2_ in a gas phase with excellent conversion values and high selectivity to produce CH_4_. The best results were obtained with the NiO with disc or sphere morphology, which produced methane with selectivity at a level near 100% and conversion close to 90%.

## 1. Introduction

Burning hydrocarbons for energy production and transport produces large amounts of carbon dioxide, negatively affecting the earth’s atmosphere [1,2]. Since the mid-18th century (industrial revolution), the amount in the earth’s atmosphere has increased by 50% [3,4]. This gas causes global warming, melting ice, and rising water levels in the world’s oceans [5,6]. Progressive electricity consumption, active motorization, and the construction industry led to an increase in the CO_2_ content in the atmosphere. The problem has increased greatly due to the reduction in forest area worldwide [7,8,9]. The Orca factory appeared in Iceland in 2021 [10,11], and the Mammoth factory was opened in Switzerland in 2024 [12]. Both factories use direct air capture (DAC) technology to condense excess CO_2_ from the atmosphere [13,14]. However, gas storage capacities will not be endless, and the question arises about the rational use of condensed gas.

CO_2_ hydrogenation seems to be a very promising process due to the relative ease of the implementation and production of various hydrocarbons [15,16]. At its core, hydrogenation is the reverse process of the combustion of hydrocarbons [17]. In the future, it will be possible to obtain a completely closed cycle—produce products by hydrogenation and use them in energy, and the resulting carbon dioxide can be used again to produce hydrocarbons.

An example of the production of hydrocarbons by the hydrogenation process is the Fischer–Tropsch reaction based on the hydrogenation of CO [18,19]. As a result of the Fischer–Tropsch reaction, a wide range of hydrocarbons can be obtained, depending on the catalyst and the chemical reaction conditions [20,21]. The same catalysts that are effective in the Fischer–Tropsch reaction (namely metals of the iron group and their oxides) are typical catalysts also for the modified Fischer–Tropsch reaction—CO_2_ hydrogenation. The simplest and most affordable hydrocarbon, methane, can be obtained in the CO_2_ methanation process [22]. As stated above, scientists are faced with the question of recycling the growing amount of CO_2_ in the earth’s atmosphere. On the other hand, the amount of natural gas is actively depleting due to its active use. CO_2_ methanation is seen as one of the most effective processes for solving both problems [17]. However, higher hydrocarbons can also be produced by the hydrogenation of CO_2_, especially using Fe- or Co-based catalysts [23,24]. The production of oxygen derivatives of hydrocarbons could be performed using other classes of catalysts; the best of them is based on In_2_O_3_ [25,26].

Nickel-based catalysts are the most efficient catalyst for producing methane by the CO_2_ hydrogenation process [27,28]. Due to its low cost and ease of production, industrial use of this catalyst is very promising. Nickel oxide can be synthesized by various methods: sonochemical synthesis [29,30], the sol–gel method [31,32], the thermal decomposition of thermally labile compounds [33,34], and, e.g., chemical vapour deposition [35,36]. Nickel oxide is very easy to obtain by the thermal decomposition of nickel hydroxide, which, in turn, can be obtained by solvothermal synthesis [37,38,39,40,41]. The solvothermal synthesis of this compound is interesting because it is not difficult to implement it in industrial chemistry. Autoclaves in which this type of reaction is carried out are similar to homogeneous synthetic reactors of chemical industries. Moreover, the solvothermal method is much cheaper and easier to realize than, for example, sonochemical and chemical vapour deposition methods. The influence of the reaction conditions on the resulting catalytic activity of the prepared catalysts is a very important standpoint, as was shown in several studies aimed at the same catalytic reaction [42,43]. Also, solvothermal synthesis enables a large variation in reaction conditions to prepare nano- and microparticles of NiO of various sizes and morphologies, significantly affecting NiO’s catalytic properties [44,45,46,47]. The catalytic activity of a substance, in particular NiO, depends crucially on the surface area of its particles. The morphology of the particles has an almost decisive influence on the surface area and, accordingly, on its catalytic activity [48,49,50].

Based on the previously published solvothermal method of synthesis of various morphologies of NiO micro- and nanoparticles, we prepared several types of them—mainly spherical and disc nano- and microparticles. They were tested subsequently as catalysts in the methanation of CO_2_ as they were not tested in this reaction previously, and they have optimal properties (especially high specific surface area) for catalytic applications. The results show that prepared NiO catalysts enable methane production from CO_2_ with a high selectivity (up to 99%) and very high conversion of CO_2_ (approximately 90%).

## 2. Experimental Section

### 2.1. Material

Ni(NO_3_)_2_·6H_2_O (98.5%), Ni(CH_3_COO)_2_·4H_2_O (Ni(Ac)_2_·4H_2_O, 98.5%), urea (99.9%), ethanol (Et, 95%), and N,N-dimethylformamide (DMF, 99.8%) were obtained from Lach-Ner, Neratovice, Czech Republic. Methanol (Met, 99.8%) and silica gel (SG) were obtained from Penta Chemicals Unlimited, Prague, Czech Republic. Polyvinylpyrrolidone (PVP, M_w_ = 40,000 and 360,000, pure), and oleylamine (98%) were purchased from Sigma Aldrich, Co. (St. Louis, MO, USA). Deionized water (DW, 18 MΩ·cm, Millipore, Burlington, MA, USA) was used to prepare aqueous solutions.

### 2.2. Catalyst Preparation

Five different methods of solvothermal synthesis in 87 mL autoclaves (Techinstro, Nagpur, India) using different solvents followed by thermal decomposition carried out in a furnace, LE 05/11 (LAC s.r.o., Židlochovice, Czech Republic), were used to prepare the NiO catalyst. Particular conditions of syntheses are summarized in Table 1.

Spherical particles NiOms1, NiOms2, and NiOms3 were prepared using methanol [51] or DMF [52,53] as a solvent. NiOms1 was synthesized from 0.2 g Ni(Ac)_2_·4H_2_O dissolved in 35 mL methanol. The solvothermal reaction was conducted at 200 °C for 4 h. After the separation of the solid product, calcination was carried out at 450 °C for 2 h. For obtaining NiOms2 and NiOms3, 0.8 g Ni(NO_3_)_2_·6H_2_O was dissolved in 70 mL DMF and kept in an autoclave under 180 °C for 12 h. Ultrasound was used for the synthesis of NiOms3 (30 W power, 15 min duration before heating). Conditions of the calcination procedure were 400 °C/4 h for obtaining NiOms2 and 350 °C/4 h for obtaining NiOms3.

NiOshc was synthesized in a mixture of oleylamine and ethanol [54]. A total of 0.5 g Ni(NO_3_)_2_·6H_2_O was dissolved in 35 mL ethanol, and after stirring, 3.5 mL oleylamine was added with an extra 17.5 mL ethanol. The reaction time in an autoclave was 15 h at 180 °C. Calcination time was 4 h at a temperature of 350 °C.

Particles with 2D morphologies were synthesized in a mixture of ethanol and water [55,56]. The syntheses of NiOhx1 and NiOhx2 morphologies were carried out in an 87 mL autoclave using Ni(NO_3_)_2_·6H_2_O-like salt and a mix of 36 mL ethanol and 4 mL H_2_O as a solvent plus PVP 40,000 and 360,000 as stabilizers [55]. Using a little less of the solvent mixture (27 mL Et + 3 mL H_2_O), a mix of hexagonal- and crescent-shaped (moon-like) morphologies (NiOhx+m) was obtained. The temperatures and reaction/calcination time were the same for all three samples—180 °C/6 h for the solvothermal reaction and 350 °C/2 h for calcination.

NiO nanodisks (NiOnd) were synthesized using the AO method described in Table 1 and in the original article [56]. A total of 0,3 gr Ni(Ac)_2_·4H_2_O was dissolved in a mixture of 22 mL ethanol and 22 mL H_2_O; the reaction in an autoclave was carried out at 200 °C/8 h. Final calcination proceeded at 450 °C/1 h in a muffle furnace.

All samples in all experiments were purified in 8 cycles (4 with water, 4 with ethanol) after the solvothermal reaction, and an additional 4 cycles were used with cyclohexane in NiOshc synthesis.

### 2.3. Instruments and Methods of Characterization

A thermogravimetric analysis was conducted with TG/DSC SDT 650 (TA Instruments, New Castle, DE, USA). The crystal structure and chemical composition of catalysts were studied by powder X-ray diffraction on the device X’pert Pro (Malvern PANalytical, Malvern, UK). The XRD analyses were carried out using a CoKa-radiation source in the 5–105° 2 Theta range and a total measurement time of 128 min/sample.

Surface area, TPR-H_2,_ and TPD-CO_2_ characterizations were measured on 3 flex Micromeritics from the Micromeritics company (Norcross, GA, USA). TPR-H_2_ and TPD-CO_2_ analyses were carried out in the temperature range of 25–600 °C with a heating rate of 10 °C/min.

The SEM images were obtained by a Scios 2 Dual Beam (ThermoFisher SCIENTIFIC, Waltham, MA, USA) microscope at an accelerating voltage of 5 kV. The TEM images were obtained by TEM JEOL 2100, 200kV (JEOL, Peabody, MA, USA).

The catalytic reactions were studied using the flow reactor Micro EFFI PID from the PID Eng & Tech company (Alcobendas, Spain). The reaction chamber was 4 mm in diameter. The analysis of reaction products was performed using Gas Chromatography with Agilent 7890B equipped with a TCD detector and Mass Spectrometer Agilent 5977B (Agilent Technologies, Santa Clara, CA, USA). The gas reaction mixture used and other conditions of the conducted reactions are stated in the next part for individual studied cases.

### 2.4. Catalytic Performance

#### 2.4.1. Parameters of Catalysis

The conversion of CO_2_ ($x_{{CO}_{2}}$, Equation (1)), selectivity of CO ($s_{CO},$ Equation (2)), selectivity of CH_4_ ($s_{{CH}_{4}},$ Equation (3)), reaction yield of CH_4_ ($\eta_{{CH}_{4}},$ Equation (4)), and space–time yield (${STY}_{{CH}_{4}}$, Equation (5)) were calculated for each catalytic system. ${STY}_{{CH}_{4}}$ indicates the amount of methane per unit weight of the catalyst.

(1) $$x_{{CO}_{2}}=\left(1-\frac{\left[{CO}_{2}\right]}{\left[{CO}_{2}\right]+\left[CO\right]+\left[{CH}_{4}\right]}\right)\times 100\%=\left(\frac{\left[CO\right]+\left[{CH}_{4}\right]}{\left[{CO}_{2}\right]+\left[CO\right]+\left[{CH}_{4}\right]}\right)\times 100\%$$

(2) $$s_{CO}=\frac{\left[CO\right]}{\left[CO\right]+\left[{CH}_{4}\right]}\times 100\%$$

(3) $$s_{{CH}_{4}}=\frac{\left[{CH}_{4}\right]}{\left[{CH}_{4}\right]+\left[CO\right]}\times 100\%$$

(4) $$\eta_{{CH}_{4}}=\frac{\left[{CH}_{4}\right]}{\left[{CH}_{4}\right]+\left[{CO}_{2}\right]+\left[CO\right]}\times 100\%$$

(5) $${STY}_{{CH}_{4}}=\frac{F_{{CO}_{2}.x_{{CO}_{2}}}s_{{CH}_{4}}}{m_{cat}}$$

$F_{{CO}_{2}}$ is the molar flow rate of CO_2_ [mmol·h^−1^], and m_cat_ is the weight of the catalyst [g].

The reaction mixture was composed of CO_2_, H_2_, and He. The pressure of the reaction mixture at the reactor was adjusted to 30 bar, and flow rates were 6 mL/min for CO_2_, 24 mL/min for H_2_, and 36 mL/min for He. The starting catalytic test was conducted with a temperature ramp from 250 up to 500 °C with a step of 50 °C (4 h of catalysis for every temperature). The optimal temperature for the catalytic stability test was determined from this experiment.

#### 2.4.2. Catalytic Tests—Temperature Ramp

**Figure 1:**
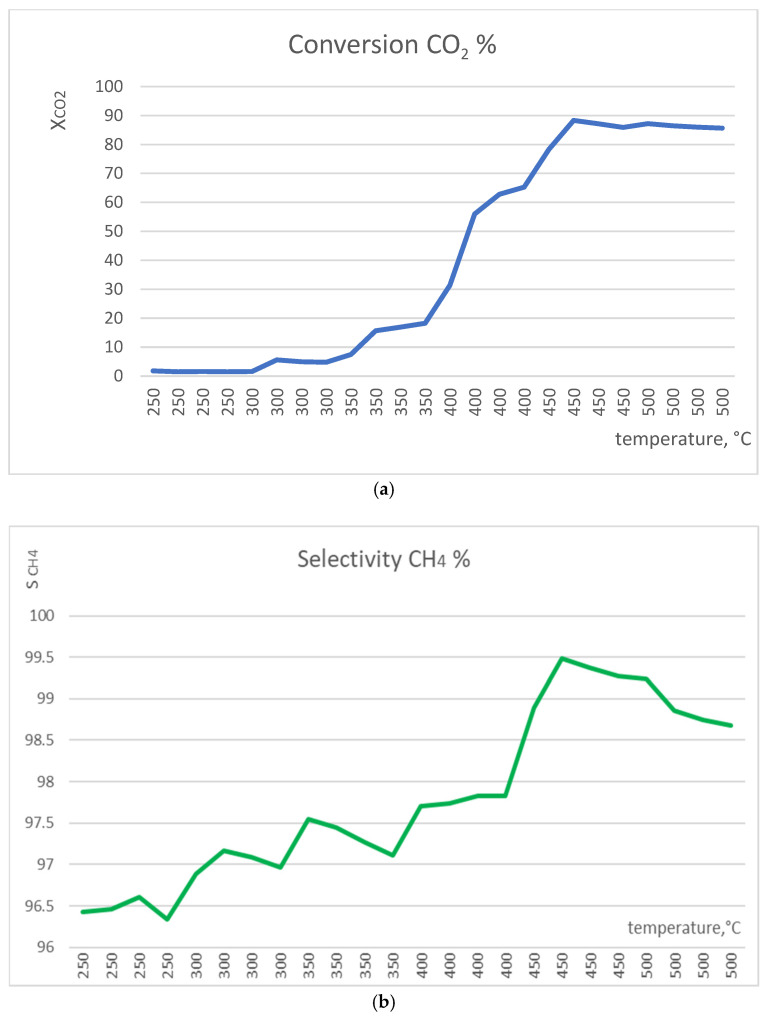
Test experiment: conversion CO_2_ (**a**) and selectivity CH_4_ (**b**) for NiOhx1 sample at temperature ramp from 250 up to 500 °C.

The solid phase of the catalytic mixture was 100 mg NiOhx1 + 150 mg SG for this test experiment. The temperature ramp used in this experiment started at 250 °C and finished at 500 °C with a 50 °C step (Figure 1). The catalytic test was conducted without any pretreatment (activation) step with the flow of CO_2_ at 6 mL/min, flow of H_2_ at 24 mL/min (ratio of H_2_ and CO_2_ of 4:1), and flow of He at 36 mL/min (55% of the gaseous mixture). The reaction pressure was adjusted to 30 bar, and the reaction proceeded at the particular temperature for 4 h after starting the temperature step.

The best conversion CO_2_ (88.35%) and selectivity CH_4_ (99.49%) were found for this catalyst at 450 °C. This temperature was chosen as optimal for all catalytic tests that were conducted. A comparison of the obtained values of conversion and selectivity with equilibrium values is presented in Figure S1.

#### 2.4.3. Catalysis Conditions

The main study of the catalytic performance of the prepared NiO catalysts was conducted under the following conditions:

(1) Used amount of catalysts’ samples—100 mg NiO + 150 mg SG.
(2) Activation of catalyst at 300 °C, 4 bars for 2 h using pure H_2_ atmosphere.
(3) Catalysis was conducted at 450 °C, 30 bar for 20 h; flow CO_2_ was adjusted to 6 mL/min and flow H_2_ to 24 mL/min (ratio of H_2_ and CO_2_ of 4:1), and flow He was 36 mL/min (55% of gaseous mixture).

A diagram showing the temperature conditions of the experiment is presented in Figure S2. However, in all presented graphs from catalytic experiments, the activation time is not taken as the initial moment of catalysis time (t = 0 h). It was taken from the moment of the end of activation and the beginning of the temperature rise to 450 °C.

## 3. Results and Discussion

**Figure 2:**
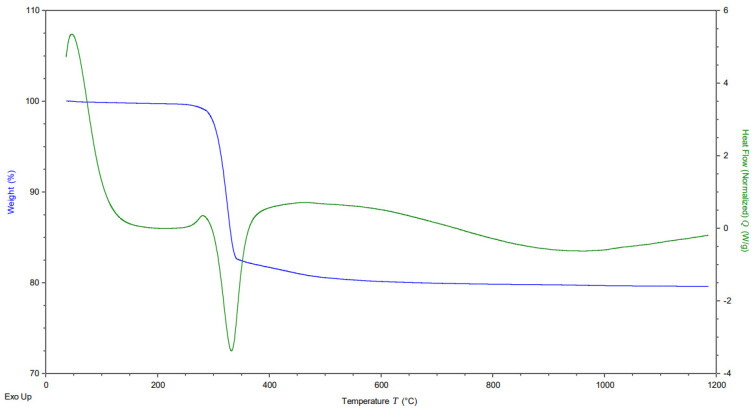
TG/DSC graphs of nickel hydroxide (pre-NiOnd, before calcination).

Nickel hydroxides prepared by the solvothermal method were calcinated after separation and purification to remove the residue of water after the drying process and to obtain a stable crystal structure of the oxide for catalytic application. The optimal calcination temperature was obtained from the thermal analysis conducted with sample Ni(OH)_2_, synthesized in conditions corresponding to the preparation of the NiOnd sample, which was used as a reference sample for this purpose. The temperature range of the analysis was 40–1200 °C (Figure 2). As can be seen on the DSC graph, the phase transformation occurs once, at about 340 degrees. The TG graph demonstrates that the sample lost about 20% of its initial mass at this temperature. XRD measurement confirmed that Ni(OH)_2_ was originally prepared and transformed into NiO by the described method. The analysis data correspond to the stoichiometric equation of the thermal decomposition of nickel hydroxide:

Ni(OH)_2_ = NiO + H_2_O (M(H_2_O)/M(Ni(OH)_2_) = 18 × 100%/92.7 = 19.41%).

XRD analyses (Figure S3) identified for all samples of the calcinated NiO only a single crystalline phase of NiO (100%) with a cubic structure and a spatial Group Fm-3m (PDF card 01-071-1179). The average size of coherent domains (MCL) varies from 9 to 19 nm for individual samples. The crystalline NiO phase is confirmed by diffraction peaks at the 2θ values of 43.5°, 50.5°, 74.5°, 90.5°, and 95.7°, corresponding to the characteristic diffraction of (111), (200), (220), (311), and (222) crystal planes of the NiO.

### 3.1. Electron Microscopy of the NiO Catalysts

#### 3.1.1. SEM/TEM before Catalysis

**Figure 3:**
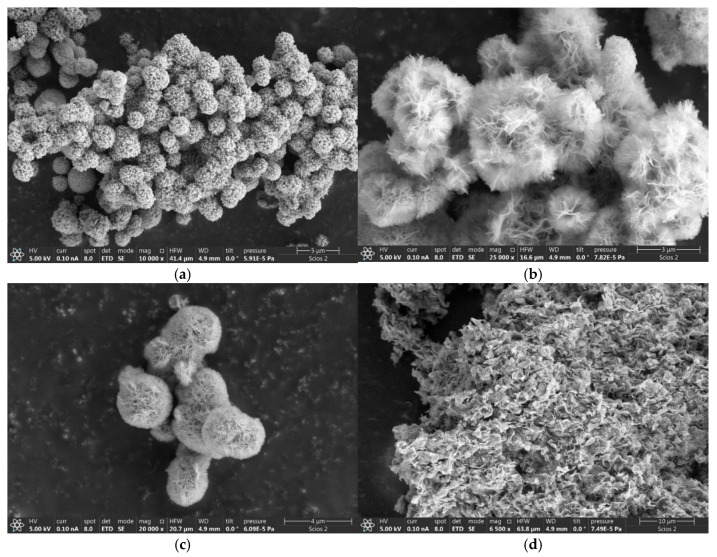

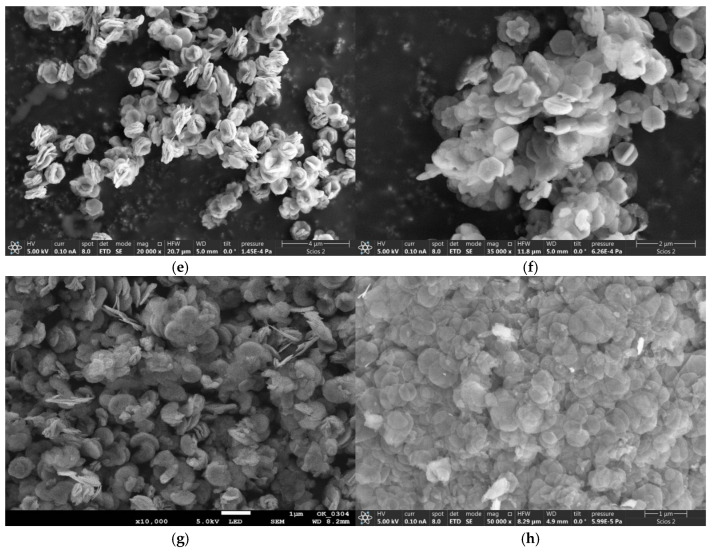
SEM images of NiO: NiOms1 (**a**), NiOms2 (**b**), NiOms3 (**c**), NiOshc (**d**), NiOhx1 (**e**), NiOhx2 (**f**), NiOhx+m (**g**), and NiO nd (**h**).

The SEM picture in Figure 3a of the NiOms1 sample shows mostly spherical microparticles of 1–3 μm in size, but shapeless ones are also found. The TEM picture (Figure S4a) shows that the spheres consist mainly of cubic crystals about 20–40 nm in size, which confirms the XRD data. SEM images in Figure 3b,c demonstrate flower-like spherical NiO MPs with a size of 1–3 μm aggregated to large clusters. However, shapeless MPs are also present in these samples. The MPs’ surface is covered with nanosheets approximately several hundred nanometers in size. In the TEM photo, it can be observed that the spheres consist of a large number of needle-like structures several hundred nanometers long and with a thickness of about 20–100 nm, which, accordingly, consist of cubic nanostructures (Figure S4b,c). The almost complete similarity of structures and sizes of MPs NiOms2 and NiOms3 is explained by identical synthesis methods; the difference is only in calcination temperature and using ultrasound during the starting part of the preparation of the NiOms3 sample. The situation in the photo (Figure 3d) is fundamentally different from previous samples: a high quantity of nanosheets with a size of about 1 micrometre are aggregated into huge clusters of tens of microns in size. Needle-like structures that are several nanometers thick and around 200 nanometers long can be traced in the TEM picture (Figure S4d). The electron microscopy observations confirmed a complete difference in morphology concerning the original article, where mesoporous microspheres were observed.

Additional preparation methods produced very similar structures, NiOhx1 and NiOhx2 (Figure 3e,f), represented by hexagonal nanoparticles of 400–600 nm in size, assembled in clusters, maybe due to the magnetism of NiO. However, NPs are visible, and the crystal faces are not coagulated together, indicating the nanoparticles’ relative stability because of using a PVP stabilizer. TEM images (Figure S4e,f) show that NPs consist of many nanocrystals, about 10 nm in size.

In Figure 3g, especially on the TEM image (Figure S4g), a large number of moon-like particles can be detected about 0.5–2 μm in size for the sample NiOhx+m. However, there are also many hexagonal structures that can be seen in the picture. NiOnd (Figure 3h) also consists of hexagonal nanodisks approximately 400–600 nm in size, which in turn consist of nanocrystals several nanometers in size, as can be observed in the TEM image (Figure S4h). However, it is clear from the SEM photo that the particles are less stable than NiOhx1 and NiOhx2 NPs and coagulate in huge clusters. It is also seen on SEM images that there are many round and shapeless fragmentary nanoparticles. As a result, it can be recognized that the hexagonal structures (NiOhx1, NiOhx2) turned out to be the most homogeneous and stable, indicating the positive role of stabilizers, as in the PVP 40,000 and 360,000.

#### 3.1.2. SEM/TEM after Catalysis

**Figure 4:**
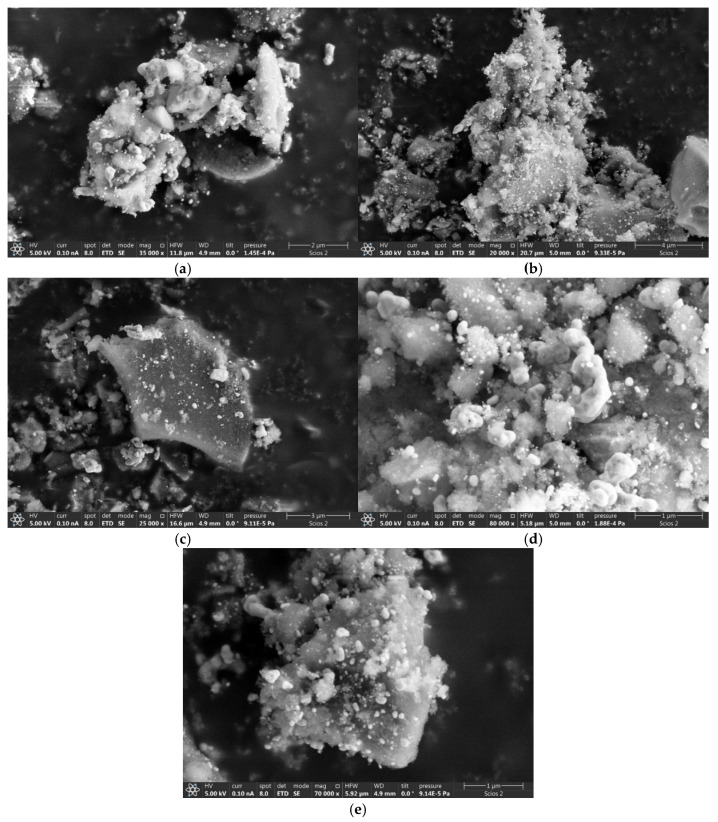
SEM images of NiO samples after catalysis: NiOms2* (**a**), NiOms3* (**b**), NiOshc* (**c**), NiOhx2* (**d**), and NiOnd* (**e**). * Marking of the spent catalyst

SEM images in Figure 4a–e reveal a cardinal change in the morphology of the NiO catalyst after the catalytic reaction. Amorphous, lumpy microparticles can be observed to be several microns in size, very different from the original particles before catalysis. It can be safely argued that this change is a result of the thermal stress and chemical modification of primary NiO particles during catalysis; particles lose their shape. However, spherical particles can be seen in the TEM pictures (Figure S5a,b), and hexagonal particles (Figure S5d,e), which indicates an incomplete loss of the morphology of the primary catalysts’ particles. All SEM pictures show brighter, whitish SG particles that did not participate in the catalytic reaction. They were used as indifferent supporting material to avoid the sintering of NiO catalytic particles.

### 3.2. Surface Area and Pore Volume Characterization

The values of the surface area (m^2^/g) and pore volume (cm^3^/g) are presented in Table 2. The NiOms1 sample has a low specific surface area—only 24.4 m^2^/g. The surface area of NiOms2 is about twice as high—47.7 m^2^/g, and NiOms3 has an essentially more developed surface—110.3 m^2^/g, which indicates ultrasound’s positive effect on microparticles’ surface. Despite the large clusters of particles, NiOshc demonstrates a very high surface area—103.7 m^2^/g, since the surface of the clusters is assembled from nano- and micro-sheets.

Samples NiOhx1 and NiOhx2 have almost the same specific surface area, which is the highest of all tested catalysts—145.5 m^2^/g. Sample NiOhx+m has about 10% less surface area (128.9 m^2^/g), indicating the appearance of many moon-like NPs. However, NiOnd has a significantly smaller specific area—55.5 m^2^/g, showing stronger nanoparticle clustering and adhesion.

Pore volumes of the prepared catalysts correlate to some extent with the values of their specific surface areas. The catalysts NiOms1, NiOms2, and NiOnd have the lowest values, the same as in the case of the surface area. Sample NiOhx1 has a much larger pore volume than sample NiOhx2 by about 30%, which indicates the positive effect of PVP 40,000 on pore volume. However, with a slight change in the synthesis conditions of NiOhx+m, the pore volume decreased significantly more than the surface area—from 0.22 to 0.16 cm^3^/g. The best value for pore volume from samples with spherical morphology was observed for NiOms3 at a value of 0.2 cm^3^/g, which can be explained by the influence of using ultrasound at the early stage of the preparation process. Graphs of measured isotherms are presented in Figure S6.

### 3.3. X-ray Photoelectron Spectroscopy

**Figure 5:**
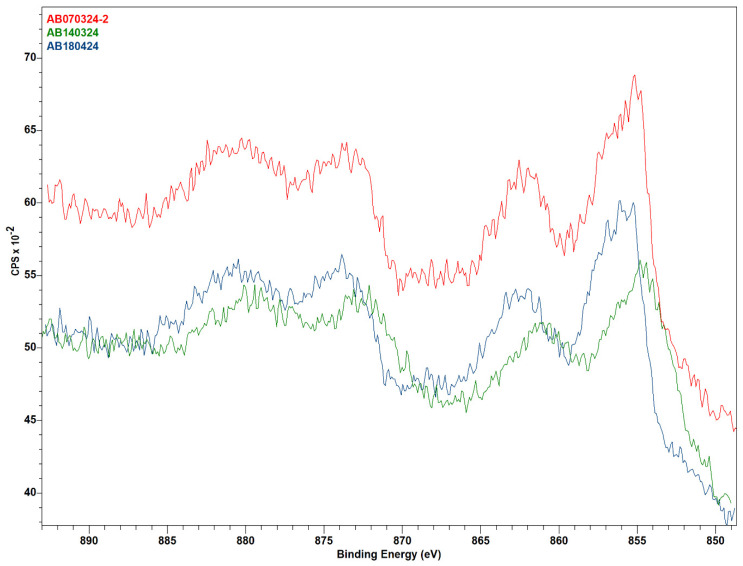
XPS (nickel 2p spectra) diagram of pre-catalysis samples: NiOhx1 (red), NiOms1 (green), and NiOhx+m (blue).

The XPS analysis (Figure 5) showed that the pre-catalysis samples have appreciable nickel content to record hi-res spectra, and there is a marked difference in NiOms1 compared to the other two. Peak fitting was attempted by assuming the nickel species present to be some mixture of NiO and Ni(OH)_2_, but the fitting was not fully successful. However, a conclusion can still be drawn from the difference in the spectra. As can be observed, the spectrum of sample NiOms1 is red-shifted off the other two, which certainly means that there is more NiO in this sample than in the other two. Sample NiOms1 was prepared after calcination at 450 °C, and the other two were calcinated at only 350 °C, which can result in a slightly larger amount of undecomposed nickel hydroxide on the surface of the catalyst. However, with all this, the catalytic efficiency of the sample NiOms1 in the CO_2_ hydrogenation was the worst.

**Figure 6:**
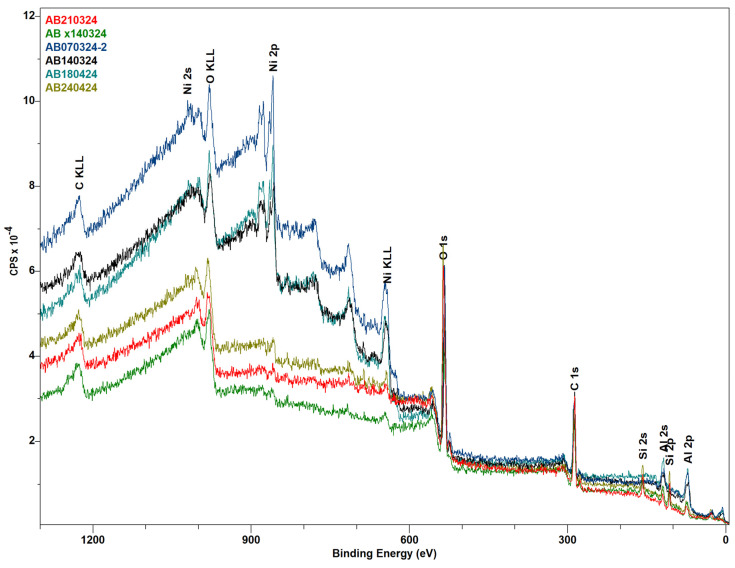
XPS (survey spectra) diagram of pre-catalysis samples, NiOms1 (black), NiOhx1 (blue), and NiOhx+m (light blue), and post-catalysis samples, NiOms1* (red), NiOhx1* (green), and NiOhx+m* (light green).

As can be seen in Figure 6 and Table 3, the XPS analysis indicated that the post-catalysis samples show small (detection-limit-adjacent) nickel content, which might stem from leaching of the metal during the reaction or, more likely, the buildup of a (mostly carbon and oxygen) “coating” on the catalyst (info depth of XPS is 5–10 nm). Really, significant differences in the spectra of all three samples, which can clarify differences in their catalytic activity, were observed after the subtraction of SiO_2_ content, which was used as an anti-sintering agent in a mixture with active catalysts. Unfortunately, the dilution of the catalysts in this inert material did not allow for the measurement of Ni’s high-resolution spectra for samples after catalysis.

### 3.4. TPR-TPD Characterization

**Figure 7:**
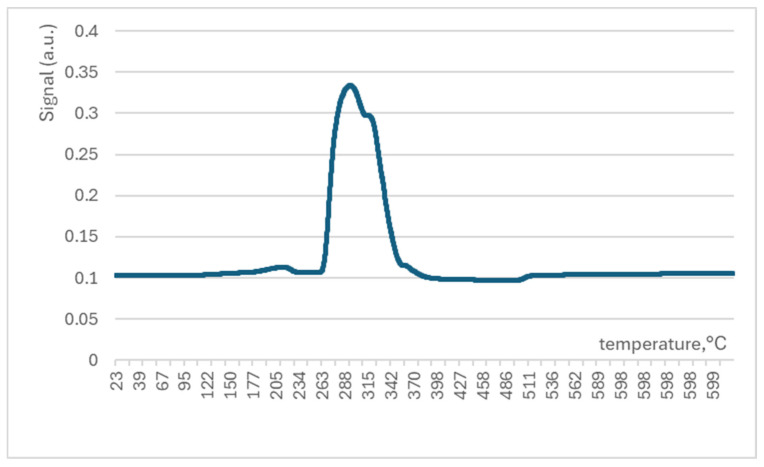
Temperature-programmed reduction (TPR-H_2_) spectrum measured with NiOhx2 sample (T = 25–600 °C).

The TPR experiment for sample NiOhx2 is presented in Figure 7, where it is possible to see the features of the interaction of nickel oxide with hydrogen. A single peak indicates the reduction of nickel oxide to pure nickel at about 300 °C. Notably, all catalysts were activated at this temperature. Therefore, reduced nickel can be supposed as the starting form of the catalyst in the reactor during the catalytic stability study.

**Figure 8:**
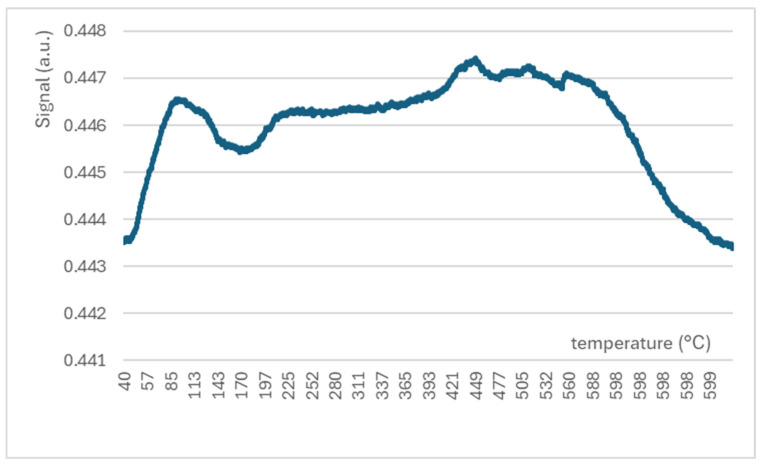
Temperature-programmed desorption (TPD-CO_2_) spectrum measured with NiOhx2 sample (T = 25–600 °C).

The TPD spectrum of the same sample is presented in Figure 8. Here, the sorption–desorption behaviour of carbon dioxide on the surface of the catalyst can be observed. The first adsorption signal is observed at about 100 °C. However, it is quickly followed by strong desorption, reaching a minimum at about 170 °C. After that, the adsorption signal of CO_2_ is raised slowly with a maximum of 450 °C. This temperature was selected as optimal for catalytic experiments also on the basis of the initial catalytic study with a temperature ramp. It is interesting that the adsorption of CO_2_ is stable from this point up to 600 °C and then steeply goes down. This behaviour indicates the chemisorption of CO_2_ on the surface of NiO-based catalysts opposite to adsorption at a lower temperature (100–170 °C), which is complementary with the mechanism of the physisorption.

### 3.5. Catalytic Reduction of CO_2_ on Different Morphologies of NiO-Based Catalysts

#### 3.5.1. NiO Disc Structures

**Figure 9:**
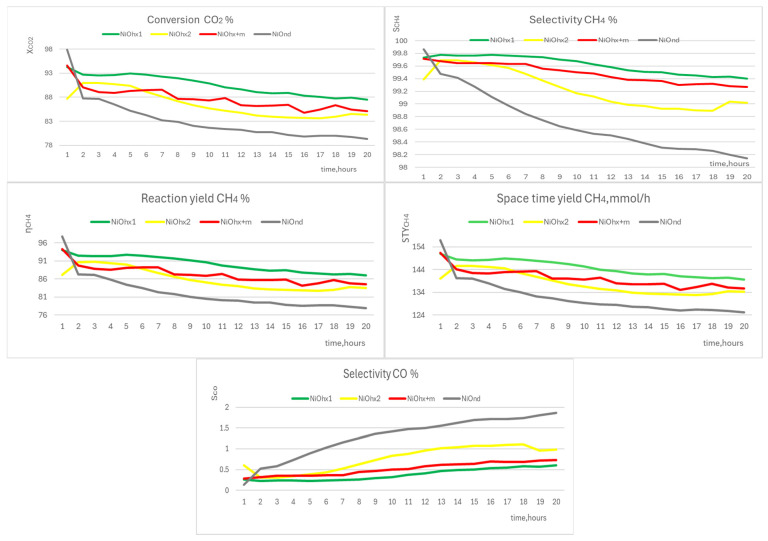
Graphs of conversion CO_2_ ($x_{{CO}_{2}}$), selectivity CH_4_ ($s_{{CH}_{4}}$), reaction yield CH_4_ ($\eta_{{CH}_{4}}$), space–time yield (${STY}_{{CH}_{4}}$), and selectivity CO (s_co_) of NiOhx1 (green), NiOhx2 (yellow), NiOhx+m (red), and NiOnd (grey).

The disc structure of NiO catalyst nanoparticles is predominant in the samples NiOhx1, NiOhx2, NiOhx+m, and NiOnd. The conversion values $x_{{CO}_{2}}$ dropped for all four samples during catalytic stability experiments, during all the 20 h experiments (see Figure 9), but the extension in this change is different for these samples. The strongest drop (more than 18%) was observed for the sample NiOnd. The total decrease was from 97.9 (t = 1 h) to 79.3% (t = 20 h), with the steepest decrease of 10% during the first 2 h of the catalytic experiment. The other three structures from this group showed a nearly linear decrease in $x_{{CO}_{2}}$ around or less than 10% for the time of the catalytic experiment (20 h) from the starting value of around 90%.

All samples demonstrated excellent selectivity for the production of methane ($s_{{CH}_{4}}$) throughout the experiment—more than 98%. The decrease in this value during the experiment was for particular catalysts as follows: NiOhx1—from 99.8 (t = 2 h) to 99.4% (t = 20 h); NiOhx2—from 99.7 (t = 2 h) to 99.0% (t = 20 h); NiOhx+m—from 99.7 (t = 1 h) to 99.3% (t = 20 h); and NiOnd—from 99.9 (t = 1 h) to 98.1% (t = 20 h). The values of $x_{{CO}_{2}}$ obtained for NiOhx1 are the best results in the selectivity and conversion of overall disc morphology catalysts. This is the sample with the highest surface area and the lowest carbon content determined by XPS. Unfortunately, the character of catalytic samples (due to dilution by SiO_2_) makes it impossible to detail the characterization of the Ni state in an active catalyst. However, it can be deduced indirectly from the results of these measurements that this type of morphology is the most resistant to the formation of atomic carbon deposits, which can hamper the access of CO_2_ molecules to the active catalytic surface and reduce, by this way, the catalytic activity of the particular catalyst.

Reaction yield ($\eta_{{CH}_{4}}$) and space–time yield (${STY}_{{CH}_{4}}$) changed according to the same pattern trend as $x_{{CO}_{2}}$ and $s_{{CH}_{4}}$. The curves of both characteristics look identical for all samples. A significant drop was observed for sample NiOnd: $\eta_{{CH}_{4}}$—from 97.8 (t = 1 h) to 77.9% (t = 20 h); and ${STY}_{{CH}_{4}}$—from 157.0 (t = 1 h) to 125.1 mmol/h (t = 20 h). The decrease in $\eta_{{CH}_{4}}$ and ${STY}_{{CH}_{4}}$ for the remaining samples was less than 10%. $\eta_{{CH}_{4}}$ values for NiOhx1, NiOhx2, and NiOhx+m samples were higher than 80% (84–87%) at the end of the experiment. ${STY}_{{CH}_{4}}$ values are also excellent, as they are in the range of 134–140 mmol/h. If we disregard the initial STY value of 157.0 mmol/h for the NiOnd catalyst, which drops sharply by about 11% to 140 mmol/h within the first two hours, then the remaining three catalysts provide significantly better results in terms of methane production. The best of these, NiOhx1, provides an STY of 151.0 mmol/h after 1 h, with a decrease to 140.0 mmol/h after 20 h of the reaction, which represents an overall decrease of only 8% compared to the NiOnd catalyst, where the decrease to 125.1 mmol/h represents a relative decrease of 17%, i.e., a value approximately twice that of the best catalyst in this group.

In all cases, the selectivity of unwanted product CO (s_CO_) increased slightly during catalysis (from 0 to 2%). The largest increase was found for the sample NiOnd—from 0.1 (t = 1 h) to 1.9% (t = 20 h). In the remaining samples, the maximum value of s_CO_ at the end of the experiment was insignificant (less than 1%)

Summing up, all four catalysts with prevailed disc morphology showed excellent results for the main indicators—selectivity CH_4_ and conversion CO_2_. Only catalyst NiOnd showed worse results in the stability of the CO_2_ conversion and the selectivity of CH_4_ production. With respect to the most complicated process of its preparation, this catalyst can be marked as the less utilizable catalyst for application in real industrial conditions.

#### 3.5.2. NiO Microsphere and Sheet-Cluster Structures

**Figure 10:**
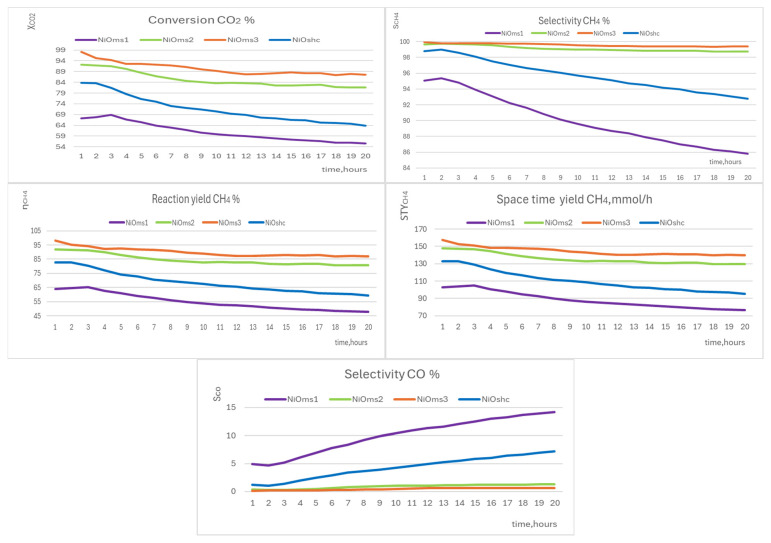
Graphs of conversion CO_2_ ($x_{{CO}_{2}}$), selectivity CH_4_ ($s_{{CH}_{4}}$), reaction yield CH_4_ ($\eta_{{CH}_{4}}$), space–time yield (${STY}_{{CH}_{4}}$), and selectivity CO (s_CO_) of NiOms1 (purple), NiOms2 (light green), NiOms3 (red), and NiOshc (blue).

The microsphere structure of NiO catalyst nanoparticles is predominant in the samples NiOms1, NiOms2, and NiOms3, while sheet-cluster morphology was observed for sample NiOshc. All samples with these types of morphology demonstrated conversion worse than was observed for the previous group of disc morphology catalytic particles (Figure 10). In particular, catalyst NiOms1 showed the worst catalytic activity of all studied catalysts. The conversion of CO_2_ declined from the value of 68.9 (t = 3 h) to the value of 55.6% (t = 20 h) in this case. This very low catalytic activity can be explained by the lowest surface area from all tested catalysts and also by the highest deposits of atomic carbon (as was determined by XPS), which can act as a catalytic barrier. The other two catalysts with microsphere morphology were in the value range of CO_2_ conversion comparable with disc morphology catalysts—in the case of NiOms2, the value of $x_{{CO}_{2}}$ declined starting from 92.3 (t = 1 h) to 81.7% (t = 20 h), and in the case of NiOms3, from 98.2 (t = 1 h) to 87.6% (t = 20 h). On the other hand, a substantial drop in the value of $x_{{CO}_{2}}$ (approx. 20%) was observed for the sample NiOshc—from 83.8 (t = 1 h) to 63.8% (t = 20 h).

The selectivity of CH_4_ production goes down almost linearly for both of the worst catalysts, NiOms1 (from 95.3 to 85.8%) and NiOshc (from 98.8 to 92.8%). The other two catalysts showed more stable selectivity for the production of CH_4_; it decreased for NiOms2 from 99.7 (t = 1 h) to 98.7% (t = 20 h) and for NiOms3 from 99.9 (t = 1 h) to 99.4% (t = 20 h). Therefore, they could be selected as the best from all the studied catalysts not only from the point of view of the selectivity production of methane but also from the point of view of ${STY}_{{CH}_{4}}$ values (see below).

Reaction yield CH_4_ ($\eta_{{CH}_{4}}$) for NiOms1 significantly dropped from 65.4 (t = 3 h) to 47.7% (t = 20 h) and for NiOshc from 82.8 (t = 1 h) to 59.2% (t = 20 h). For NiOms2 and NiOms3 samples, $\eta_{{CH}_{4}}$ dropped a bit more than 10%; for NiOms2, it dropped from 91.9 (t = 1 h) to 80.6% (t = 20 h) and for NiOms3 from 98.1 (t = 1 h) to 87.0% (t = 20 h). ${STY}_{{CH}_{4}}$ changed in all four samples in a similar way, but for the two worst catalysts, the decrease in this value with time is more considerable. The biggest drop was observed for the NiOms1 catalyst, from 105.0 (t = 3 h) to 76.6 mmol/h (t = 20 h), and also for the second worst catalyst, the NiOshc decrease was considerable as ${STY}_{{CH}_{4}}$ was changed from 132.9 (t = 1 h) to 95.0 mmol/h (t = 20 h). On the other hand, the two best catalysts in this group, NiOms2 and NiOms3, showed 157.5 mmol/h (NiOms3) or 147.6 mmol/h (NiOms2) at the start reaction with a slow decrease of about 10% (140 mmol/h for NiOms3 after 20 h) or 12% (130 mmol/h for NiOms2 after 20 h).

Selectivity CO increased very much for NiOms1, from 4.9 (t = 1 h) to 14.2% (t = 20 h). NiOms2 and NiOms3 showed non-important increases in s_CO_ from 0.3 (t = 2 h) to 1.3% (t = 20 h, NiOms2) and from 0.1 (t = 1 h) to 0.6% (t = 20 h, NiOms3). In the case of NiOshc, which is the second worst catalyst from all those tested, s_CO_ increased much more from 1.0 (t = 2 h) to 7.2% (t = 20 h).

To sum up, the two best catalysts from this group are those prepared in the DMF solution—NiOms2 and NiOms3. Due to the strong drop in conversion CO_2_ for catalysts NiOms1 and NiOshc, industrial use of catalysts does not seem promising.

#### 3.5.3. Comparison of Catalytic Activity of Prepared NiO Structures

The solvothermal method used for the preparation of the NiO catalyst showed good variability in the morphology of the prepared catalysts by simply changing the used solvent and alternatively using a polymer stabilizing agent. The results obtained from the catalytic activity of these NiO-based catalysts are very good in comparison with those of more sophisticated catalysts in Table 4. The presented data clearly show that simple NiO-based catalysts with optimal morphology can produce methane with efficiency comparable to Ni catalysts modified or supported by the more valuable metals prepared using more complicated and time-consuming methods. The only disadvantage of the newly prepared catalysts is that their optimal catalytic activity requires a relatively high temperature of 450 °C, which increases the energy consumption of the catalytic process in practise. However, the simple composition of the catalyst offers future possibilities to optimize the reaction temperature, e.g., by modification with active support instead of the simple dilution of the NiO catalyst by the inert micro-SiO_2_.

The observed high catalytic activity of the prepared pure NiO catalyst does not only come from their high specific surface area, which is more than about 140 m^2^/g for the best catalysts NiOhx1, NiOhx2, and NiOhx+m and 110 m^2^/g for NiOms2 and NiOms3. Another important property is given by the nature of their nanostructured surface, thanks to which, in the activation phase, NiO can be partially transformed into metallic Ni, which is essential in terms of the activation of hydrogen molecules for their own reaction with the carbon dioxide molecule [57]. The resulting metallic nickel nanoparticles are also detectable in XPS spectra. Here, it can be seen that for active catalysts, there is a signal of metal Ni in the spectrum at the energy level of 852.5 eV. Unfortunately, this signal is not well quantifiable due to the low concentration of Ni in the used catalyst caused by the use of SiO_2_ as an auxiliary material. On the other hand, the gentle decrease in the catalyst activity observed during the tests cannot be ascribed to the formation of the carbon deposits on the surface of the catalysts as neither XPS spectra nor SEM images in the secondary electron mode (see Figure S7) proved the existence of relevant carbon deposits.

**Table 4:** The basic results of the catalytic action of the prepared catalysts compared with the literature data.

Catalyst	Reaction Temperature (°C)	$x_{{CO}_{2}}$ (%)	$s_{{CH}_{4}}$	Ref.
NiOms3	450	91	99	this work
NiOhx1	450	91	99	this work
La-doped Ni/SiO_2_	275	71	99	[58]
Ni/Y_2_O_3_	340	55	60	[59]
Ni/Y_2_O_3_	300	92	100	[60]
Ni/ITQ6 zeolite	450	79	98	[61]
Ni/MgO	275	79	98	[62]
Ni/CeO_2_	400	80	98	[63]
Ni/CeO_2_	420	94	100	[64]
Ni/perovskite	425	61	98	[65]

## 4. Conclusions

Based on the experiments conducted, the following important conclusions can be drawn:

1. Catalysts prepared by simple solvothermal methods using inexpensive and green solvents (water, ethanol), namely NiOhx1, NiOhx2, and NiOhx+m, demonstrated excellent catalytic activity in CO_2_ methanation, especially due to their well-developed surface area (145 m^2^/g) and good resistance to blocking of the active catalyst area by deposits of atomic carbon, which is a problem of many methanation catalysts.
2. NiO catalytic samples prepared using the solvothermal method in the DMF solvent (NiOms2 and NiOms3) also showed very good catalytic activity, especially NiOms3, since it has a more developed surface (110 m^2^/g), which may be due to the use of ultrasound in synthesis. However, the use of an expensive DMF and not green solvent and a long reaction time make the preparation of this catalyst more expensive than the preparation of the above-mentioned disc structures.
3. NiOnd, NiOms1, and NiOshc are very poor catalysts due to a high drop in methane conversion and methane selectivity during the catalytic reaction (20 h). The results of XPS measurements also indicate lower resistance of these structures against deposits of atomic carbon during the reaction course. This fact is probably the main reason for the observed time instability of the catalytic efficiency of these catalysts. Therefore, the modifications of the solvothermal method used for the preparation of these catalysts are not promising for advanced studies.

## Figures and Tables

**Table 1:** Summary table of synthesis conditions of prepared catalysts.

Sample	Abbrev.	Ni Salt	Solvent	Stabilizer	Tsynt, °C	Tsynt, h	Tcalc, °C	Tcalc, h
NiO microspheres 1	NiOms1	Ni(Ac)_2_	Met	---	200	4	450	2
NiO microspheres 2	NiOms2	Ni(NO_3_)_2_	DMF	---	180	12	400	4
NiO microspheres 3	NiOms3	Ni(NO_3_)_2_	DMF	--- Ultrasound	180	12	350	4
NiO sheet clusters	NiOshc	Ni(NO_3_)_2_	Et	Oleylamine	180	15	350	4
NiO hexagonal 1	NiOhx1	Ni(NO_3_)_2_	Et + DW	PVP 40,000	180	6	350	2
NiO hexagonal 2	NiOhx2	Ni(NO_3_)_2_	Et + DW	PVP 360,000	180	6	350	2
NiO hexagonal + moon-like	NiOhx+m	Ni(NO_3_)_2_	Et + DW	PVP 40,000	180	6	350	2
NiO nanodisk	NiOnd	Ni(Ac)_2_	Et + DW	---	200	8	450	1

Tsynt, °C—solvothermal reaction temperature; tsynt, h—solvothermal reaction time; Tcalc, °C—calcination temperature of nickel hydroxide; tcalc, h—calcination time.

**Table 2:** Surface area and pore volume of NiO samples.

Sample Name	NiOms1	NiOms2	NiOms3	NiOshc	NiOhx1	NiOhx2	NiOhx+m	NiOnd
BET surface area, m^2^/g	24.4	47.7	110.3	103.7	145.5	145.5	128.9	55.5
Pore volume, cm^3^/g	0.049	0.113	0.202	0.166	0.221	0.169	0.162	0.123

**Table 3:** The content of elements on the surface of the catalyst before and after (*) catalysis.

Sample	Ni	Ni ^#^	O	O ^#^	C	C ^#^	Si
NiOms1*	1.2	2.0	39.3	21.6	46.4	76.5	13.2
NiOhx1*	1.2	1.5	39.2	32.2	52.8	66.3	6.8
NiOhx1	11.0	---	35.7	---	53.4	---	---
NiOms1	10.9	---	38.1	---	51.0	---	---
NiOhx+m	11.3	---	41.6	---	47.1	---	---
NiOhx+m*	1.6	2.6	43.9	29.3	41.5	68.0	13.1

All values are presented in atomic %. ^#^ denotes content of element after subtraction of SiO_2_ content in catalytic mixture.

## Data Availability

The original contributions presented in the study are included in the article/Supplementary Material, further inquiries can be directed to the corresponding author.

## Supplementary Materials

The following supporting information can be downloaded at: https://www.mdpi.com/article/10.3390/molecules29204838/s1, Figure S1: Comparison of the experimental and equilibrium values for the methanation of CO_2_ obtained in the test experiment with NiOhx1 sample. Figure S2: Diagram describing temperature change with time during catalytic experiments. Figure S3: XRD patterns of NiO pre-catalysts (CoKa-radiation). Figure S4: TEM images of NiO pre-catalysts. Figure S5: TEM images of NiO samples after catalysis. Figure S6: Isotherm plot and BET surface area plot diagrams of NiO pre-catalysts. Figure S7: SEM images of the spent catalyst samples obtained in secondary electrons mode.
