# Supplementary material for: NiO Nano- and Microparticles Prepared by Solvothermal Method—Amazing Catalysts for CO2 Methanation
Source: Molecules. 2024 Oct 12;29(20):4838. doi: 10.3390/molecules29204838 (PMC11510583; doi:10.3390/molecules29204838)
Supplement: Supplementary file 1 [file molecules-29-04838-s001.zip › molecules-3186271-supplementary.pdf]

Supplementary Information

NiO Nano- and Microparticles Prepared by Solvothermal Method—Amazing Catalysts for CO_2 Methanation

Arkadii Bikbashev [1], Tomáš Stryšovský [1], Martina Kajabová [1], Zuzana Kovářová [1], Robert Prucek [1], Aleš Panáček [1], Josef Kašlík [2], Tamás Fodor [3], Csaba Cserháti [4], Zoltán Erdélyi [4] and Libor Kvítek [1,*]

[1] Department of Physical Chemistry, Faculty of Science, Palacky University Olomouc, 17. listopadu 12, CZ-77146 Olomouc, Czech Republic; arkadii.bikbashev01@upol.cz (A.B.)

[2] Czech Advanced Technology & Research Institute CATRIN, Regional Centrum of Advanced Technologies & Materials, Palacký University Olomouc, Slechtitelu 27, CZ-78371 Olomouc, Czech Republic

[3] HUN-REN Institute for Nuclear Research, H-4002 Debrecen, Hungary

[4] Department of Solid-State Physics, Faculty of Sciences and Technology, University of Debrecen, H-4002 Debrecen, Hungary; zoltan.erdelyi@science.unideb.hu (Z.E.)

* Correspondence: libor.kvitek@upol.cz; Tel.: +420-585-634-420

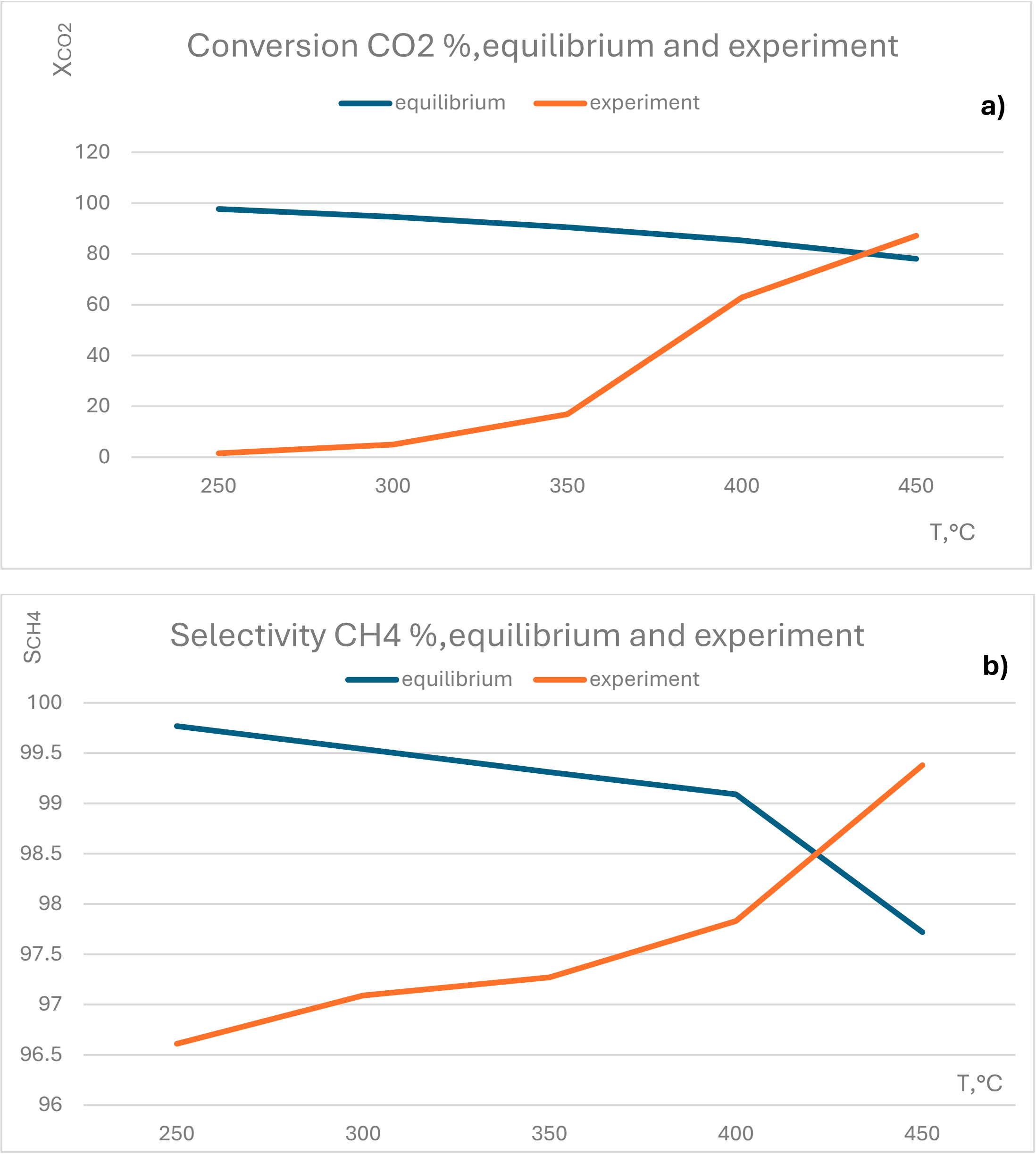

Figure S1. Comparison of the experimental and equilibrium values for the methanation of CO2 obtained in the test experiment with NiOhx1 sample.

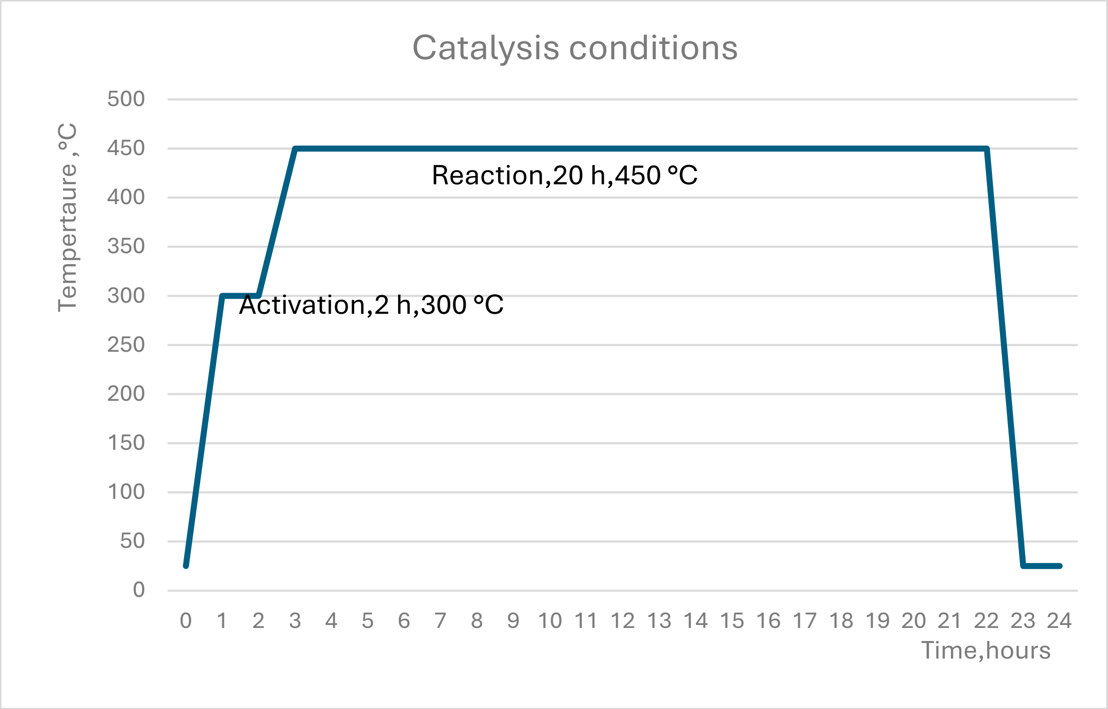

Figure S2. Diagram describing temperature change with time during catalytic experiments.

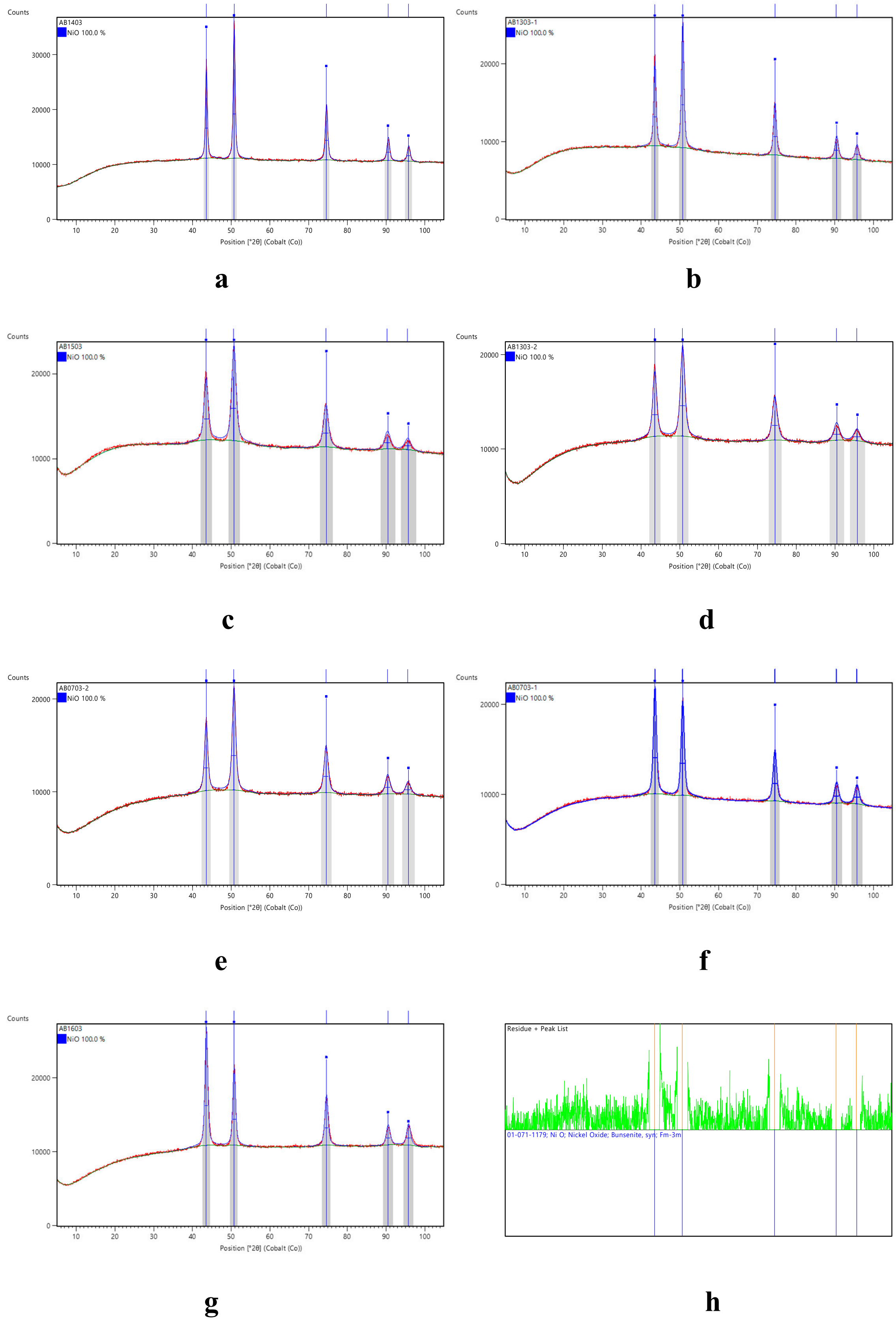

Figure S3. XRD patterns of NiO pre-catalysts (CoKa-radiation): NiOms1 (a), NiOms2 (b), NiOms3 (c) NiOshc (d), NiOhx1 (e), NiOhx2 (f), NiOnd (g), and map Fm-3m group NiO Bunsenite (h).

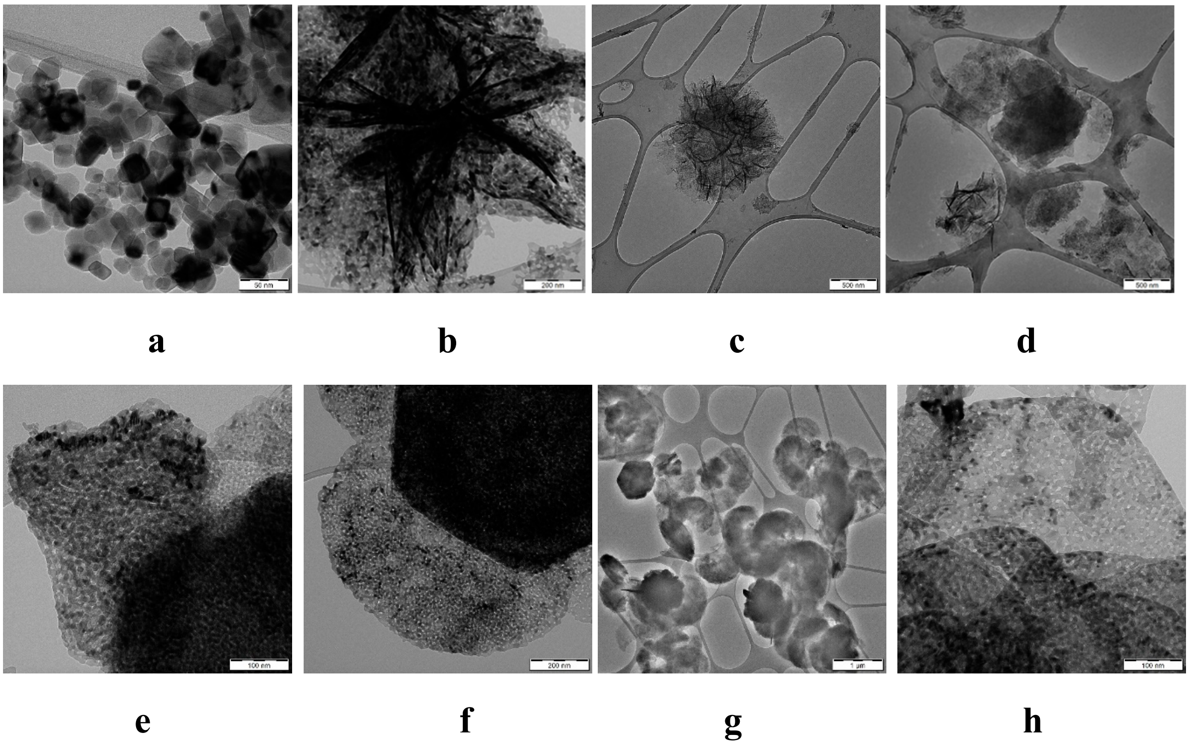

Figure S4. TEM images of NiO pre-catalysts: NiOms1 (a), NiOms2 (b), NiOms3 (c), NiOshc (d), NiOhx1 (e), NiOhx2 (f), NiOhx+m (g), and NiOnd (h).

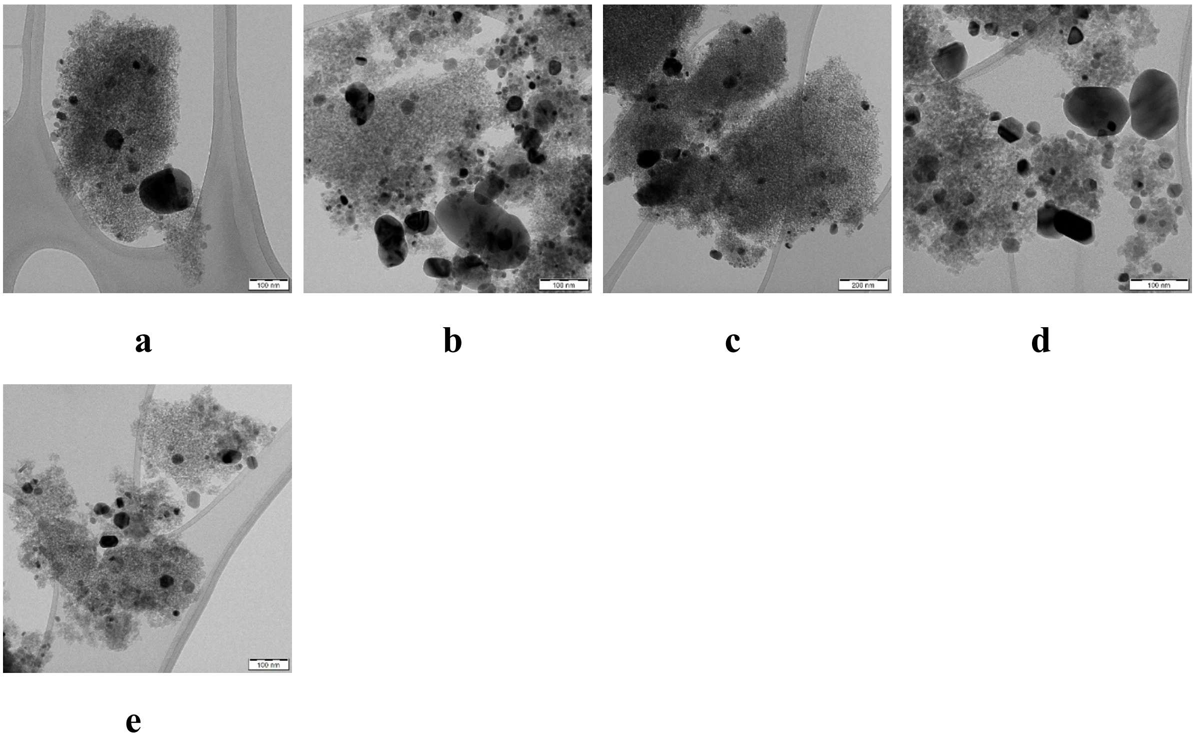

Figure S5. TEM images of NiO samples after catalysis: NiOms2 (a), NiOms3* (b), NiOshc* (c), NiOhx2* (d) and NiOnd* (e).*

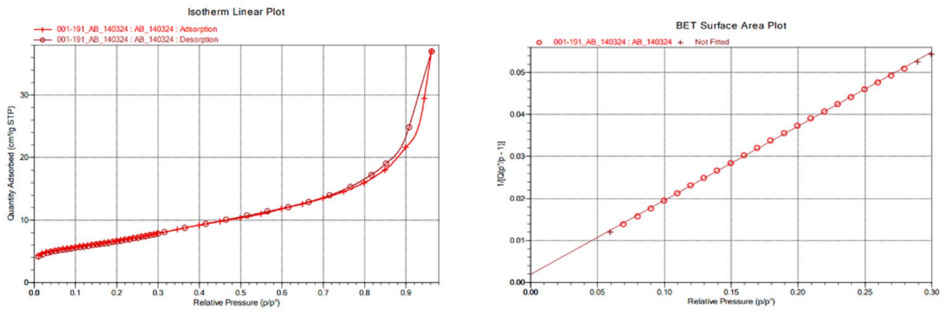
Isotherm Linear Plot
001-191_AB_140324 : AB_140324 : Adsorption
001-191_AB_140324 : AB_140324 : Desorption
Quantity Adsorbed (cm³/g STP)
Relative Pressure (p/p°)
BET Surface Area Plot
001-191_AB_140324 : AB_140324
Not Fitted
1/[Q(p°/p - 1)]
Relative Pressure (p/p°)

a

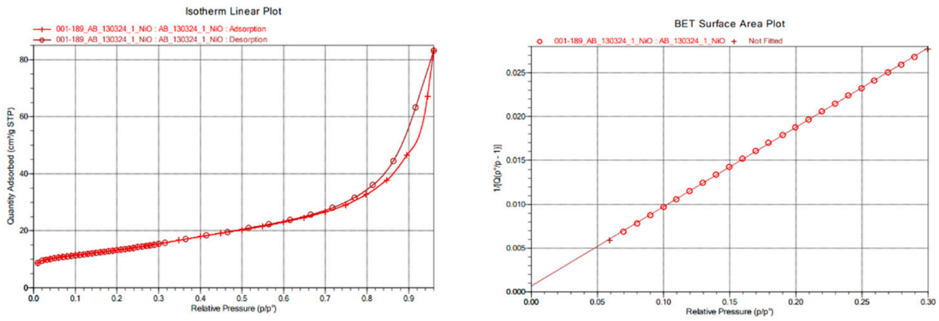
Isotherm Linear Plot
001-189_AB_130324_1_NiO : AB_130324_1_NiO : Adsorption
001-189_AB_130324_1_NiO : AB_130324_1_NiO : Desorption
Quantity Adsorbed (cm³/g STP)
Relative Pressure (p/p°)
BET Surface Area Plot
001-189_AB_130324_1_NiO : AB_130324_1_NiO
Not Fitted
1/[Q(p°/p - 1)]
Relative Pressure (p/p°)

b

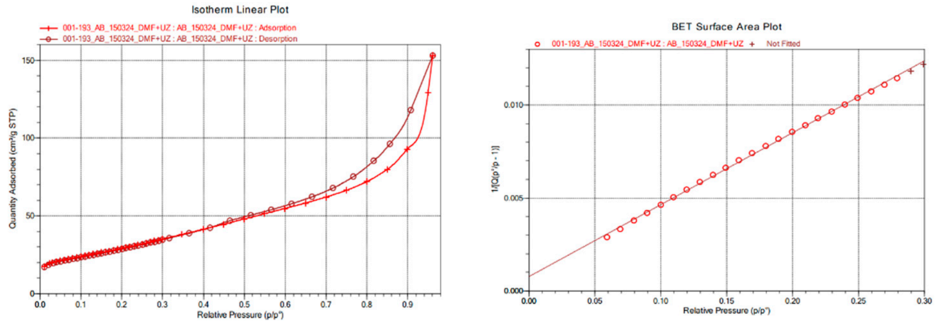
Isotherm Linear Plot
001-193_AB_150324_DMF+UZ : AB_150324_DMF+UZ : Adsorption
001-193_AB_150324_DMF+UZ : AB_150324_DMF+UZ : Desorption
Quantity Adsorbed (cm³/g STP)
Relative Pressure (p/p°)
BET Surface Area Plot
001-193_AB_150324_DMF+UZ : AB_150324_DMF+UZ
Not Fitted
1/[Q(p°/p - 1)]
Relative Pressure (p/p°)

c

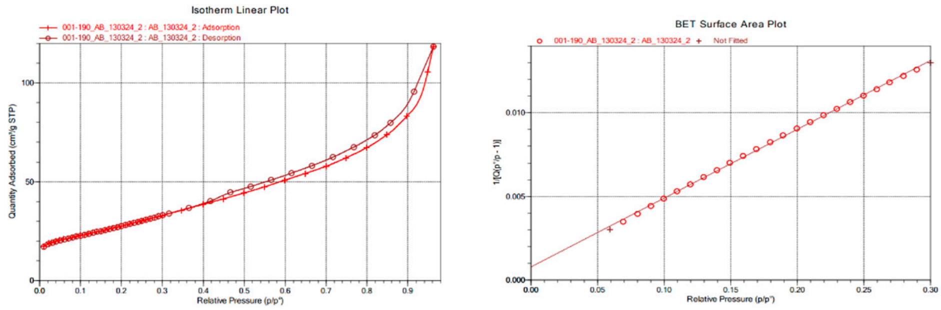
Isotherm Linear Plot
001-190_AB_130324_2 : AB_130324_2 : Adsorption
001-190_AB_130324_2 : AB_130324_2 : Desorption
Quantity Adsorbed (cm³/g STP)
Relative Pressure (p/p°)
BET Surface Area Plot
001-190_AB_130324_2 : AB_130324_2
Not Fitted
1/[Q(p°/p - 1)]
Relative Pressure (p/p°)

d

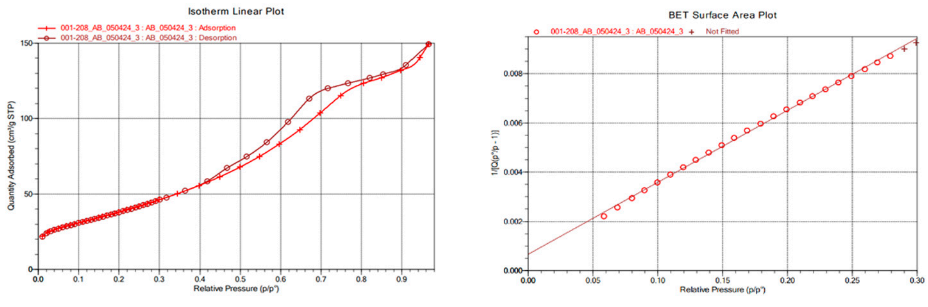
Isotherm Linear Plot
001-208_AB_050424_3 : AB_050424_3 : Adsorption
001-208_AB_050424_3 : AB_050424_3 : Desorption
Quantity Adsorbed (cm³/g STP)
Relative Pressure (p/p°)
BET Surface Area Plot
001-208_AB_050424_3 : AB_050424_3
Not Fitted
1/[Q(p°/p - 1)]
Relative Pressure (p/p°)

e

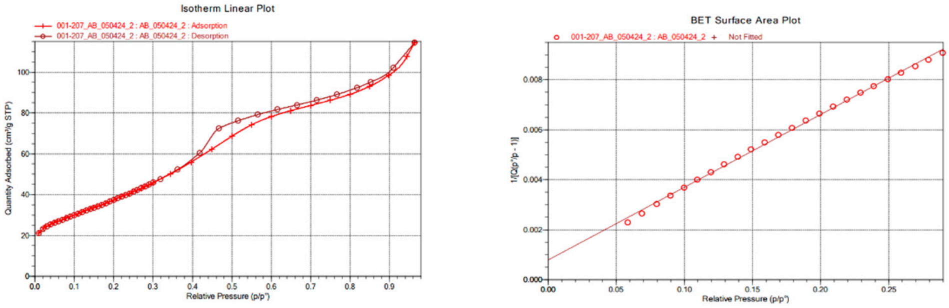

f

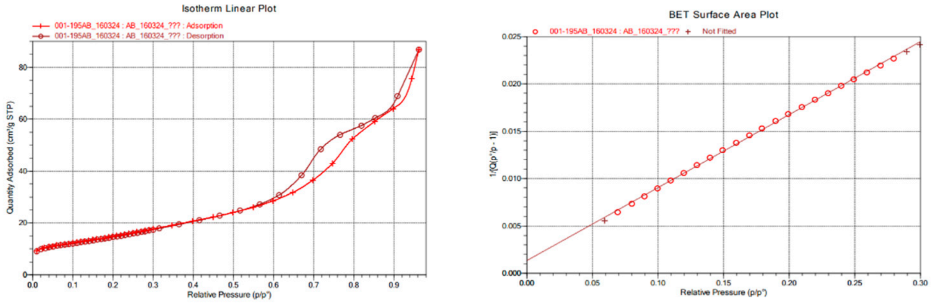

g

Figure S6. Isotherm plot and BET surface area plot diagrams of NiO pre-catalysts: NiO$_{ms1}$ (a), NiO$_{ms2}$ (b), NiO$_{ms3}$ (c), NiO$_{shc}$ (d), NiO$_{hx1}$ (e), NiO$_{hx2}$ (f), and NiO$_{nd}$ (g).

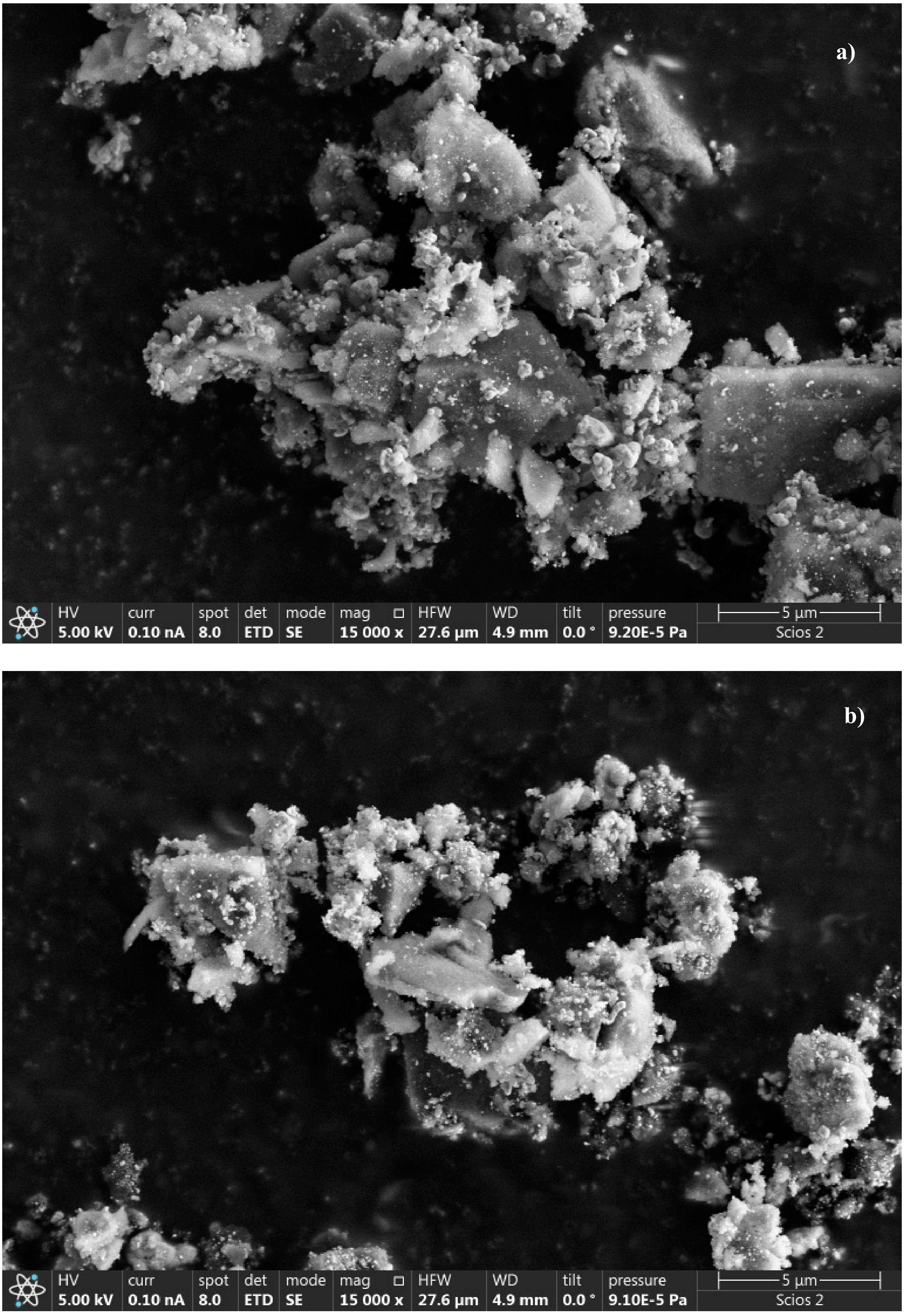

Figure S7. SEM images of the spent catalyst samples obtained in secondary electrons mode. Catalysts NiOhx2 (a) and NiOms3 (b).
